# Crossing the Barrier: *Eikenella corrodens* Bacteremia Following CNS Infection in a Patient Treated with Nivolumab—A Case Report and Literature Review

**DOI:** 10.3390/microorganisms13092135

**Published:** 2025-09-12

**Authors:** Terenzio Cosio, Cataldo Maria Mannavola, Barbara Fiori, Matteo Zelinotti, Francesco Taccari, Brunella Posteraro, Tiziana D’Inzeo, Maurizio Sanguinetti

**Affiliations:** 1 Department of Basic Biotechnological Sciences, Intensive and Perioperative Clinics, Università Cattolica del Sacro Cuore, 00168 Rome, Italy; cataldomannavola5@gmail.com (C.M.M.); brunella.posteraro@unicatt.it (B.P.); 2 Department of Laboratory and Hematological Sciences, Fondazione Policlinico Universitario A. Gemelli IRCCS, 00168 Rome, Italy; barbara.fiori@policlinicogemelli.it (B.F.); matteo.zelinotti@policlinicogemelli.it (M.Z.); tiziana.dinzeo@unicatt.it (T.D.); 3 Infectious Diseases Unit, Department of Medical and Surgical Sciences, Fondazione Policlinico Universitario A. Gemelli IRCCS, 00168 Rome, Italy; francesco.taccari@policlinicogemelli.it

**Keywords:** *Eikenella corrodens*, brain abscess, bacteremia, immune checkpoint inhibitors, nivolumab, oral microbiota, neoplasms, sepsis, dysbiosis, head and neck neoplasms

## Abstract

*Eikenella corrodens* is a facultative anaerobic Gram-negative bacillus, part of the normal oropharyngeal flora, with opportunistic pathogenic potential particularly in immunocompromised hosts. The progression from localized intracranial infections such as cerebritis and subdural empyema to secondary bloodstream infection represents a rare but clinically significant pathway, especially in immuno-compromised patients. Here, we report a case of secondary *E. corrodens* bacteremia following left temporal cerebritis and ipsilateral subdural empyema in a 50-year-old man with advanced nasopharyngeal carcinoma treated with nivolumab. The patient presented neurological deficits and systemic inflammatory response, suggesting for a bacterial infection. Neuroimaging confirmed the intracranial infectious foci and blood cultures identified *E. corrodens* via MALDI-TOF MS and 16S rRNA gene sequencing. We discuss how cancer-associated immune dysregulation and immune checkpoint inhibition could modulate host susceptibility and clinical presentation of infection, potentially facilitating microbial dissemination across compromised blood–brain barriers. Additionally, we examine the cases of *E. corrodens* bacteremia secondary to CNS and head and neck infections. This case underscores the importance of heightened clinical vigilance for secondary bacteremia in oncologic patients with CNS infections and highlights the need for integrated microbiological and radiological assessment to optimize outcomes.

## 1. Introduction

Infections involving the brain, spinal cord, optic nerves, and meninges-collectively known as central nervous system (CNS) infections are among the most debilitating conditions associated with significant morbidity, mortality, and lasting complications [1]. CNS infections are classified according to the predominant anatomic site of involvement: the meninges (meningitis), brain parenchyma (encephalitis or brain abscess), or spinal cord (myelitis), although significant overlap frequently occurs in syndromes such as meningoencephalitis or encephalomyelitis [2]. Among the various pathogenic routes, contiguous spread from adjacent head and neck infections represents a particularly important mechanism, second only to hematogenous dissemination [3]. Infections arising from the head and neck region pose a unique clinical challenge, given the anatomical complexity and constant exposure of mucosal surfaces to polymicrobial flora. Conditions affecting this region—ranging from benign inflammatory disorders to malignancies—are frequently complicated by local and systemic infectious processes, particularly when mucosal integrity is disrupted [4,5,6]. Infections of the head and neck may, through contiguous extension from dental, otic, or sinonasal foci, give rise to rare but potentially fatal intracranial complications such as cerebritis, brain abscess, or subdural empyema [7,8]. In particular, paranasal sinusitis frequently underlies the formation of temporal or frontal lobe abscesses or subdural collections, depending on the involved sinus cavity and route of extension [9]. These infections typically present with nonspecific neurological symptoms and imaging findings, complicating early diagnosis—especially in patients with complex underlying conditions [10]. Although CNS infections are traditionally considered compartmentalized processes, disruption of the blood–brain barrier (BBB) may, in rare cases, facilitate microbial translocation into the bloodstream, leading to secondary bacteremia.

This phenomenon assumes particular relevance in cancer patients, whose immunological milieu is often altered not only by the neoplastic process itself but also by the cumulative impact of therapeutic interventions such as chemotherapy, radiotherapy, and emerging immunomodulatory agents including immune checkpoint inhibitors (ICIs) like nivolumab [11,12].

While ICIs enhance anti-tumor immunity by blocking inhibitory pathways such as programmed cell death (PD)-1/PD-L1 and cytotoxic T-lymphocyte-associated (CTLA) protein-4, they can induce a state of immune dysregulation rather than classical immunosuppression. This dysregulated immunity may paradoxically increase susceptibility to infections through several mechanisms. Firstly, ICIs promote nonspecific T-cell activation, potentially disrupting immune homeostasis and impairing effective immune surveillance against pathogens, thereby facilitating opportunistic infections and reactivation of latent microbes. Secondly, neuroinflammatory effects associated with ICIs and underlying malignancy may compromise BBB integrity, allowing pathogens to breach CNS defenses and disseminate systemically. Thirdly, adjunctive treatments common in these patients, such as corticosteroids used to manage immune-related adverse events (irAEs), confer additional immunosuppressive effects that further elevate infection risk. Lastly, ICIs may indirectly alter mucosal immunity and microbiota composition, promoting overgrowth and translocation of commensal organisms like *Eikenella corrodens* [13]. This organism is a facultative anaerobic, fastidious Gram-negative bacillus that resides as a commensal in the oral, gastrointestinal, and genitourinary mucosa. It belongs to the HACEK group of organisms—an acronym referring to *Haemophilus* spp., *Aggregatibacter* spp., *Cardiobacterium hominis*, *Eikenella corrodens*, and *Kingella* spp.—which are known for their fastidious growth requirements and association with culture-negative endocarditis [14,15,16]. Although *E. corrodens* is among the less commonly isolated members of the group, it has been increasingly recognized as a clinically relevant pathogen in polymicrobial infections of the head and neck, CNS, and bloodstream, particularly in the setting of mucosal barrier breakdown or immunocompromised hosts [14,15,16]. Its low intrinsic virulence is often masked by diagnostic delay, while its variable antibiotic susceptibility profile can hinder effective empirical therapy. Here, we describe a case of *E. corrodens* bacteremia secondary to left temporal cerebritis and subdural empyema in a patient with advanced nasopharyngeal carcinoma undergoing nivolumab therapy. This case serves as a basis for a broader review of the literature on *E. corrodens* infections of head and neck origin, highlighting their potential for intracranial extension and systemic dissemination, particularly in immunologically vulnerable hosts.

## 2. Case Report

A 50-year-old Caucasian man, former smoker (4–5 cigarettes/day for approximately 10 years; quit in 2014), with a history of Epstein–Barr virus (EBV)-positive, non-keratinizing undifferentiated squamous cell carcinoma (SCC) of the nasopharynx, initially diagnosed in April 2023 and staged as cT4N1M0 (Stage IVa), presented to our center with acute onset of high-grade fever (temperature 39 °C), altered mental status, severe aphasia, a new right oculomotor nerve palsy, and predominantly brachial right-sided hemiparesis not previously noted in the emergency department. The patient had recently initiated immunotherapy with nivolumab (240 mg IV every two weeks, since March 2025), with the most recent dose administered three days prior to symptom onset, in the context of leptomeningeal progression. The patient’s oncologic course had been marked by an aggressive disease trajectory. Initial treatment consisted of concurrent chemoradiotherapy (total dose 70–63–56 Gy over 35 fractions) and cisplatin (cumulative dose 185 mg/m^2^), which was discontinued prematurely after two cycles due to digital ischemia of the second digit of the left hand from radial artery thrombosis. In May 2023, surgical resection of a right nasopharyngeal lesion confirmed poorly keratinizing SCC, followed by adjuvant brachytherapy (3000 cGy, 250 cGy BID). Despite these measures, the patient experienced clinical and radiologic progression between August 2023 and May 2024. A biopsy performed in June 2024 confirmed recurrence of EBV-positive, non-keratinizing SCC (p40+), and systemic chemotherapy with cisplatin plus gemcitabine was initiated, resulting in partial metabolic response on PET after six cycles. From October 2024, gemcitabine maintenance monotherapy was pursued. Serial plasma EBV DNA quantification showed fluctuating levels, with upward trends noted in January and March 2025, suggestive of molecular progression. At the time of current presentation, the patient was functionally impaired, immunologically altered by both underlying malignancy and recent immunotherapy, and at risk for atypical or opportunistic infections.

**Figure 1:**
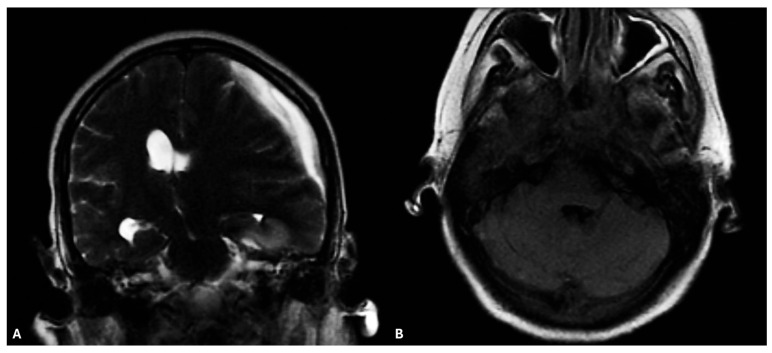
(**A**) Brain RM revealed Left hemispherical supratentorial subdural fluid layer, without hypointense hemorrhagic components in SWI in context, with a maximum thickness of 17 mm (vs. 14 mm), presenting with peripheral contrast enhancement and diffusion restriction, similar to empyema. Increased mass signs with compression of the left ventricle, and an 11 mm (vs. 9 mm) displacement of the septum pellucidum to the right, with apparent left uncial progression. Right ventricular dilation (due to incarceration), with T2W/FLAIR hyperintensity at the level of the posterior horn, in a context of hydrocephalus due to obliteration of the foramina of Monro. (**B**) Evaluation of the facial structures demonstrated extensive mucosal edema involving the sphenoidal sinuses and ethmoidal labyrinths, suggestive of an underlying sinonasal infection with possible contiguous spread to the intracranial compartment. No signs of acute intracranial hemorrhage or hydrocephalus were observed. These findings raised concern for cerebritis and incipient subdural empyema. Technique: 1.5 T scanner, FSPGR, FSE, SWAN, 3D-FLAIR, and DWI sequences in multiple planes before and after intravenous administration of paramagnetic contrast medium (ProHance 0.2 mL/kg).

At the admission, initial vital signs revealed a blood pressure of 110/60 mmHg, heart rate of 110 beats per minute, and oxygen saturation of 100%. Laboratory investigations demonstrated normocytic anemia (hemoglobin 10.7 g/dL; hematocrit 33.6%; MCV 83.2 fL), marked neutrophilic leukocytosis (white blood cell count 13.49 × 10^9^/L; neutrophils 12.54 × 10^9^/L), platelet count of 328 × 10^9^/L, and normal coagulation (INR 1.08). Renal function was preserved (blood urea nitrogen 11 mg/dL; creatinine 0.81 mg/dL; estimated glomerular filtration rate 107 mL/min/1.73 m^2^ by CKD-EPI), with normal serum electrolytes (sodium 137 mmol/L; potassium 3.6 mmol/L). An urgent diagnostic workup was initiated, suspicious of local infection in a contest of disease progression, including blood and urine cultures, chest radiography, and brain computed tomography (CT). Brain CT revealed a left temporal hypodensity associated with hemispheric swelling and midline shift, while the evaluation of the facial mass showed extensive edema of the sphenoidal sinuses and ethmoidal labyrinths, suggestive of an infection of the same. Empiric antimicrobial therapy with intravenous piperacillin–tazobactam 18 g/d was promptly started. Subsequent brain magnetic resonance imaging (MRI), obtained following neurosurgical consultation, demonstrated features consistent with left temporal cerebritis accompanied by an ipsilateral subdural empyema. These findings were suggestive of contiguous spread of infection from adjacent structures, particularly the sphenoidal sinuses and ethmoidal labyrinths (Figure 1). Serial neuroimaging confirmed progressive enlargement of the subdural collection. Moreover, considering signs and symptoms, a concomitant bloodstream infection (BSI) was suspected, and peripheral venous and central venous catheter (CVC) blood cultures were collected on the day of admission. Paired aerobic and anaerobic bottles were incubated using the BACT/ALERT^®^ Virtuo^®^ automated system (bioMérieux, Marcy l’Étoile, France). After 28 h, the blood cultures resulted positive and following the definitive microbiological diagnosis, based on culture, MALDI TOF MS and 16S rRNA gene sequencing, *E. corrodens* bloodstream infection was detected. Considering the antimicrobial susceptibility profile, piperacillin–tazobactam was escalated to intravenous ceftriaxone, administered at a dosage of 2 g once daily. The patient exhibited progressive clinical improvement, with defervescence within 48 h, normalization of leukocyte counts, and resolution of inflammatory markers. Two follow-up blood-culture sets obtained 48 and 72 h after ceftriaxone initiation remained negative, indicating microbiological clearance. A multidisciplinary evaluation was undertaken involving infectious diseases, neurology, oncology, and neurosurgery teams. Given the extent of the intracranial involvement—characterized by temporal cerebritis and ipsilateral subdural empyema in the context of an immunocompromised host—and the overall limited prognosis due to underlying metastatic squamous cell carcinoma of the nasopharynx, neurosurgical intervention was deemed inappropriate. The decision was based on the absence of a clear indication for surgical drainage, lack of neurological improvement, and anticipated limited benefit in the setting of advanced oncologic disease. Moreover, after reevaluating the treatment plans already performed (70 Gy with photons as first-line therapy and a subsequent 30 Gy delivered as brachytherapy), the possibility of performing hadrontherapy was ruled out due to both the high risk of radiation necrosis and the impossibility of performing hadrontherapy with a palliative dose.

Following a structured process of shared decision-making with the patient’s family, a palliative approach was adopted. The patient was transitioned to comfort-focused care and subsequently transferred to a residential hospice facility for ongoing symptom management and end-of-life support.

## 3. Materials and Methods

### 3.1. Microbiological Culture and Microscopy

Following bottle positivity, aliquots were sub-cultured onto appropriate solid media, including Chocolate Blood Agar (PVX), MacConkey Agar, Tryptic Soy Agar (TSA), Schaedler Agar and Columbia CNA Agar (bioMérieux, Marcy l’Étoile, France), and incubated under aerobic and microaerophilic conditions at 35–37 °C. Colony growth was assessed after 24–48 h. In parallel, Gram staining for morphological evaluation was performed by light microscopy (Olympus, Carl Zeiss, Cambridge, UK) at 20× and 100× magnification.

### 3.2. MALDI-TOF MS Identification of E. corrodens

The acquisition and analysis of mass spectra were performed using the MALDI Biotyper^®^ (Bruker Daltonics, Bremen, Germany) system, operated with the MALDI Biotyper software MBT Compass 4.1, the standard Bacterial Library (Bruker Daltonics, Bremen, Germany), under default parameter settings as recommended by the manufacturer. Calibration was performed using the Bruker Bacterial Test Standard (BTS) in accordance with the manufacturer’s protocol. A small amount of a fresh bacterial colony was applied directly onto a stainless steel MALDI target plate (MTP 384; Bruker Daltonics, Bremen, Germany). Subsequently, 1 µL of α-cyano-4-hydroxycinnamic acid (HCCA; Bruker Daltonics) matrix solution was added to each spot and air-dried. The target was then analyzed using the Bruker Biotyper system, and spectra were matched against the manufacturer’s reference database for identification. The MALDI Biotyper software (version 3.4) compared each acquired mass spectrum with the reference spectra in the database, assigning a score ranging from 0.00 to 3.00 based on pattern similarity. Identification was interpreted using standard Bruker score criteria: scores ≥ 2.0 were accepted for species-level identification, scores between 1.7 and <2.0 indicated genus-level identification, and scores < 1.7 were considered unreliable. The software also reported consistency categories—A (species-level consistency), B (genus-level consistency), and C (no consistency)—which were recorded for interpretative support. In addition, identification robustness was evaluated by retrospectively lowering the species cutoff to 1.9, 1.8, and 1.7, and the genus cutoff to 1.6 and 1.5, with reassessment of the top 10 database matches to explore borderline identifications.

### 3.3. Molecular Confirmation of E. corrodens Identification

To confirm the identity of the isolate previously identified by MALDI-TOF MS as *E. corrodens*, partial 16S rRNA gene sequencing was performed. Genomic DNA was extracted from pure bacterial cultures using the EZ2 Connect (Qiagen, Hilden, Germany) in accordance with the manufacturer’s instructions. PCR amplification targeted a variable region of the 16S rRNA gene using the universal bacterial primers 27F (5′-AGAGTTTGATYMTGGCTCAG-3′) and 787R (5′-GGACTACCAGGGTATCTAAT-3′). The reaction mixture consisted of 10 µL of 2× PCR Mix, 2 µL of each primer (10 pmol/µL), 1 µL of DNA template, and 5 µL of nuclease-free water, for a final volume of 20 µL. Amplification was carried out in a GeneAmp 9700 PCR System (Applied Biosystems, Foster City, CA, USA) with standard thermal cycling conditions: initial denaturation at 95 °C for 3 min, followed by 35 cycles of denaturation at 95 °C for 30 s, annealing at 55 °C for 30 s, and extension at 72 °C for 1 min, concluding with a final extension at 72 °C for 5 min. Amplicons were purified using the MiniElute Gel Extraction Kit (Qiagen, Hilden, Germany) and sequenced using the BigDye Terminator Kit v3.1 (Applied Biosystems). Sequencing was performed on a SeqStudio™ Genetic Analyzer System (Applied Biosystems), and resulting chromatograms were analyzed using Sequence Analysis Software v5.0. The consensus sequence was compared to reference sequences in the NCBI nucleotide database using the BLASTn algorithm to confirm species-level identity.

### 3.4. Antimicrobial Susceptibility Testing of E. corrodens

Antimicrobial susceptibility testing of the *E. corrodens* isolate was performed using E-test^®^ (bioMérieux, Marcy-l’Étoile, France) gradient diffusion strips according to the manufacturer’s instructions. Briefly, a standardized bacterial suspension equivalent to a 0.5 McFarland standard was prepared in sterile saline and inoculated onto Mueller–Hinton agar supplemented with 5% defibrinated sheep blood and 20 mg/L β-NAD (bioMérieux, Marcy l’Étoile, France), in accordance with recommendations for fastidious organisms. Plates were incubated at 35 ± 2 °C in a 5% CO_2_ atmosphere and examined after 24 and 48 h. The minimum inhibitory concentrations (MICs) were read at the point of complete inhibition of visible growth. Since neither EUCAST nor CLSI provide species-specific breakpoints for *E. corrodens*, MICs were reported descriptively without categorical interpretation.

### 3.5. Study Research and Inclusion Strategies

We performed a comprehensive search of the following databases from 1958 to June 2025: Cochrane Central Register of Controlled Trials; MEDLINE; Embase; US National Institutes of Health Ongoing Trials Register; NIHR Clinical Research Network Portfolio Database; and the World Health Organization International Clinical Trials Registry Platform. Reference lists and published systematic review articles were analyzed. We used the terms “*Eikenella corrodens*” and/or “*Bacteroides corrodens*” with the following keywords, separately and in combination: “sepsis”, “septicemia”, “bacteremia”, “bloodstream infection”, “head”, “brain” and “neck”. Only English-language articles were included in the search. Forward citation analyses of original studies and review articles were also conducted.

In all the studies in which other bacteria or fungi isolated from patients, we considered only the results related to *E. corrodens* bacteremia and where a head-and-neck source with contiguous spread was identified. All human studies were included with no age, sex, ethnicity, or type of scientific study-related restriction. We considered only case reports and case series in which *E. corrodens* was a pathogen causing first head and neck infections and a secondary bacteremia and all case reports that have not yet been included in reviews or clinical trials.

Exclusion criteria were articles discussing non-CNS and non-head and neck *E. corrodens* infection followed by bacteremia (e.g., endocarditis, pleural empyema, intra-abdominal or vertebral infections) and non-English-language publications.

## 4. Results

### 4.1. Blood Culture Isolation and Microscopic Examination of E. corrodens

**Figure 2:**
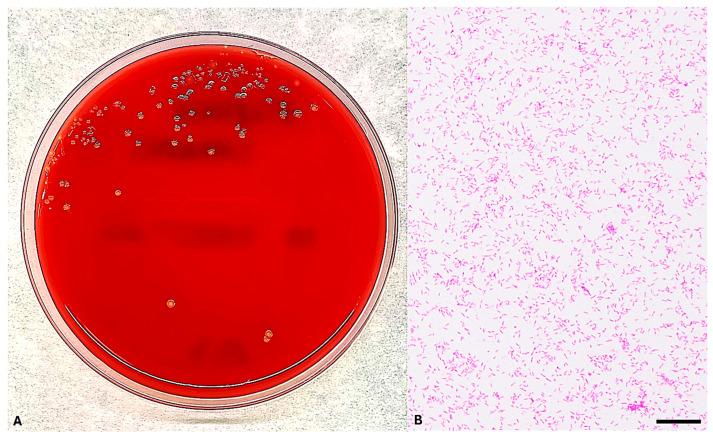
(**A**) On TSA incubated under aerophilic conditions at 37 °C, *Eikenella corrodens* formed small, convex, grayish-yellow, translucent to opaque colonies with a characteristic pitting of the agar surface. (**B**) Gram staining revealed slender, pleomorphic, Gram-negative rods arranged singly or in short chains, without spore formation. Scale bar, 10 µm.

With clinical suspicion of bloodstream infection (BSI), blood cultures were obtained on the day of hospital admission from both peripheral venous (PV) access and a central venous catheter (CVC). Paired aerobic and anaerobic blood culture bottles were inoculated and incubated using the BACT/ALERT^®^ Virtuo^®^ blood culture system (bioMérieux, Marcy l’Étoile, France). After 28 h of incubation, two aerobic bottles from peripheral venous draws flagged as positive. Gram staining of the subcultures revealed slender, rod-shaped Gram-negative bacteria. The isolate exhibited growth on TSA and formed small, translucent grayish-yellow colonies with pitting of the agar surface on blood and chocolate agar under 5% CO_2_ at 37 °C after 48 h, consistent with the morphological characteristics of *E. corrodens* (Figure 2). The organism was subsequently identified by MALDI-TOF MS and confirmed through partial 16S rRNA gene sequencing.

### 4.2. MALDI-TOF MS-Based Identification of E. corrodens

Identification of the isolate was performed using MALDI-TOF MS with the Bruker Biotyper^®^ system (Bruker Daltonics, Bremen, Germany). A high-confidence identification of *E. corrodens* was obtained, with score values ranging from 2.03 to 2.11 across replicate analyses, consistent with reliable species-level identification according to Bruker interpretative criteria (≥2.0 for species-level assignment). No conflicting identifications were recorded among the top ten database matches.

### 4.3. Sequencing-Based Identification of E. corrodens

To definitively confirm the species identification of the bloodstream isolate, genomic DNA was extracted and subjected to PCR amplification targeting the 16S rRNA gene using primers 27F and 787R. The resulting amplicon was sequenced bidirectionally, and the consensus sequence was analyzed via BLAST against the NCBI nucleotide database (https://blast.ncbi.nlm.nih.gov/Blast.cgi accessed on 5 May 2025). The sequence analysis revealed a 100% identity match with *E. corrodens* reference NCTC10596, confirming the species-level identification initially obtained by MALDI-TOF MS. The 16S rRNA gene sequence has been deposited in GenBank under the accession number PV598151.

### 4.4. MIC Results of E. corrodens

The minimum inhibitory concentrations (MICs) for the clinical *E. corrodens* isolate are reported in Table 1. No organism-specific interpretive criteria for antimicrobial susceptibility testing of *E. corrodens* are currently defined by either the Clinical and Laboratory Standards Institute (CLSI) or the European Committee on Antimicrobial Susceptibility Testing (EUCAST). The isolate displayed low MICs indicative of in vitro susceptibility to beta-lactams such as ceftriaxone, penicillin G, and imipenem, as well as to macrolides, lincosamides, and folate pathway inhibitors. Taken together, the antimicrobial profile suggests a broadly susceptible phenotype.

### 4.5. Literature Review of E. corrodens Secondary Bacteremia

A total of 54 articles concerning *E. corrodens* infection were identified according to the predefined search strategy. Of these, 47 were excluded after applying the initial exclusion criteria (non-human studies, reviews, or cases unrelated to invasive infection). Among the remaining seven reports, two were further excluded after abstract and full-text evaluation because they described *E. corrodens* bacteremia arising from sources outside the head, neck, or intracranial compartments (e.g., endocarditis, abdominal sepsis). Consequently, five articles were retained for inclusion in this review. The decision to restrict the analysis to head and neck or intracranial infections was based on the clinical relevance to our case and on the specific pathophysiological pathway of interest, namely, the contiguous spread from local infections across disrupted mucosal and anatomic barriers, with subsequent progression to bloodstream dissemination. Within this framework, a review of published cases (Table 2) provides valuable clinical insights into the spectrum of *E. corrodens* infections arising from head and neck sources and their potential intracranial complications followed by bacteremia [17,18,19,20,21].

In 1978, Emmerson and Mills [17] described a fatal case of meningitis and right cerebellar abscess, followed by sepsis, in a 60-year-old immunocompetent woman with a history of repeated head and neck surgeries. This was the first report of a fatal case due to *E. corrodens* originated from a head and neck infection. In 1992, Zgheib and colleagues [18] reported a 52-year-old man who developed systemic infection following a peritonsillar abscess; he recovered following combination therapy in a *E. corrodens* infection exhibiting clindamycin and amikacin resistance. In 2013, Dong and Gong [19] detailed a case of chronic meningitis in a 50-year-old woman with alcoholism and smoking history, managed successfully with ampicillin–sulbactam and levofloxacin. A case of early-onset sepsis with associated meningitis due to *E. corrodens* in a 27-week preterm neonate was described by Sawyer et al. [20] in 2015; broad-spectrum empirical antibiotics led to survival. Lastly, Yamagishi et al. [21] reported a case of Lemierre’s syndrome in a 44-year-old man with IgG4-related ophthalmic disease and immunosuppression, in whom *E. corrodens* and *Gemella bergeri* were co-isolated from blood cultures. These cases highlight *E. corrodens* as a pathogen capable of causing intracranial infection via local extension from oropharyngeal or sinonasal sites, particularly in settings of anatomical disruption (e.g., surgery, malignancy, mucosal breach) or systemic vulnerability (e.g., cancer, immunosuppression, prematurity). Moreover, cases reviewed in Table 2 highlight several features of *E. corrodens* infection in patients with head and neck disease. The symptoms of these infections are various and include a plethora of features starting from fever, respiratory distress, and dysphagia arriving to behavior disturbs. Looking at all reported cases, we can assume that *E. corrodens* infection frequently is a benign infection; the poor outcome synergistic role of the patient’s comorbidities, and thus subsequent BSI occurs as the final blow [17]. *E. corrodens* BSI is also a widespread infection: it is present in almost every state, and all ethnicities are affected; at the same time, both infants and adults can be affected by *E. corrodens* BSI, suggesting how the important commensal role of this bacterium could rapidly switch into dangerous pathogen [18,20]. Risk factors evidenced to develop for *E. corrodens* BSI are dental surgery procedures, head and neck pathologies, oral dysbiosis due to many factors, smoke and immunocompromised state, including immunomodulant therapies [18,21]. Fortunately, significant antimicrobial resistance in *E. corrodens* infections have not been revealed, with susceptibility to many antibiotics used in empiric and de-escalation therapies. As in previously published cases, the clinical presentation of our case was characterized by non-specific systemic and focal neurological manifestations—fever, altered mental status, and focal neurological deficits—underscoring the consistent lesson that early neuroimaging and prompt microbiological investigation are pivotal for accurate diagnosis. Nonetheless, several aspects render our case distinctive and warrant critical emphasis. First, the host context: unlike most reports, which primarily describe immunocompetent adults or isolated neonatal cases, our patient was an adult with advanced EBV-positive nasopharyngeal squamous cell carcinoma undergoing treatment with nivolumab. The close temporal proximity between ICI initiation and clinical deterioration raises the possibility that recent immune modulation may have contributed to altered mucosal immunity, barrier dysfunction at the skull base, or shifts in local microbial ecology facilitating bacterial invasion. However, causality cannot be inferred from temporal association alone. Accordingly, our observation should be regarded as hypothesis-generating rather than definitive evidence of an ICI-mediated predisposition. This data suggests also the need for a rapid diagnosis in critical and at-risk patients, allowing prompting antibiotics administration for *E. corrodens* bacteremia.

## 5. Discussion

*Eikenella corrodens* is a facultative anaerobic Gram-negative bacillus belonging to the HACEK (*Haemophilus*, *Aggregatibacter*, *Cardiobacterium*, *Eikenella*, and *Kingella*) group, typically residing as a commensal organism in the oral cavity and upper respiratory tract [22]. Under normal circumstances, it rarely causes invasive infections. However, mucosal disruption or alteration of the resident microbiota can facilitate its transition from colonizer to pathogen, leading to infections ranging from head and neck abscesses to endocarditis and bacteremia [23]. In our case, the initial intracranial infection likely originated from contiguous spread of *E. corrodens* from the sphenoidal and ethmoidal sinuses to the adjacent temporal lobe, culminating in cerebritis and subdural empyema. Anatomical continuity between sinonasal structures and intracranial spaces, facilitated by thin bony barriers and venous communications, is a well-documented pathway for such infections [24]. The progression from localized CNS infection to systemic bacteremia signifies a breach of multiple protective barriers, compounded by the patient’s oncological condition and, potentially, immunotherapy. Immune checkpoint inhibitors (ICIs) such as nivolumab have revolutionized cancer treatment by reinvigorating T-cell mediated anti-tumor immunity through PD-1 receptor blockade [25]. However, this immunomodulation can paradoxically predispose patients to infections. A comprehensive meta-analysis of randomized clinical trials encompassing more than 21,000 patients reported an incidence of all-grade infections of approximately 9.6% in ICI-treated arms versus 8.3% in controls, while grade 3–5 infections occurred in 3.1% of ICI recipients compared with 2.6% in control groups [26]. Data from registrational trials consistently document infections among the most frequent adverse events associated with ICIs, particularly upper respiratory tract infections, pneumonia, and urinary tract infections, often exceeding the 10% threshold in frequency. For instance, upper respiratory infections have been reported in 12–20% of patients [27]. Real-world evidence further underscores the clinical significance of these findings. In retrospective cohorts of patients with lung cancer receiving ICIs, severe infections requiring hospitalization or intravenous antibiotics were not uncommon, with concomitant high-dose corticosteroid use emerging as a key risk factor [28,29,30,31]. Several mechanisms could be potentially implicated in the risk infection during ICIs treatment. First, ICIs may dysregulate immune homeostasis, leading to immune-related adverse events (irAEs) that can cause inflammation and damage to anatomical barriers, including the blood–brain barrier [32]. Second, T-cell exhaustion reversal might alter the balance of immune responses, impair effective pathogen clearance and facilitate microbial invasion. While the intestinal microbiota has been extensively studied, emerging evidence suggests that mucocutaneous microbiota, including the oral microbiome, plays a crucial role in maintaining barrier immunity and preventing pathogen colonization [32,33]. Although data are still limited, recent studies have begun to characterize shifts in oral microbial communities during ICI treatment, documenting decreased microbial diversity and the emergence of pathogenic species such as *Candida* and *Streptococcus* spp. [34,35,36,37]. So, immunotherapy could potentially disrupt the mucosal barrier and alter the host microbiome, resulting in dysbiosis and enhanced pathogenic potential of commensal bacteria such as *E. corrodens*. This altered microbial ecology, coupled with immune system alteration, may enable bacteria to penetrate mucosal surfaces, invade contiguous tissues, and enter the bloodstream [32]. Notably, oral bacteria translocation into the bloodstream could be caused also by normal oral hygiene activity, like chewing and toothbrushing and dental therapeutic procedures too [38]. This could determine both transient bacteremia or infective consequences like endocarditis in at risk patients [39]; indeed, studies report bloodstream presence of oral bacteria as *Porphyromonas gingivalis*, *Aggregatibacter actinomycetemcomitas* and *E. corrodens* in patients after periodontal therapies [39]. Our patient’s clinical presentation with neurological deficits, fever, and laboratory evidence of systemic inflammation prompted rapid imaging and microbiological investigation, essential steps given the nonspecific clinical features and fastidious nature of *E. corrodens*. Longitudinal monitoring of the microbiota—before, during, and after immunotherapy—could provide valuable insights into treatment-related dysbiosis and its clinical implications. Pre-treatment microbiota profiling has already been proposed as a predictive biomarker for response to ICIs [40], while dynamic monitoring might allow early detection of dysbiosis linked to adverse events or infections.

Moreover, diagnosis of *E. corrodens* infection is challenging due to its slow growth and special culture requirements. In this case, blood cultures processed with the BACT/ALERT^®^ Virtuo^®^ system became positive after 28 h, and MALDI-TOF MS provided rapid and reliable bacterial identification. Molecular confirmation via 16S rRNA gene sequencing further validated the diagnosis. In this context, molecular techniques, such as Nested PCR and Real-Time PCR, performed directly on blood samples, may support the diagnosis of bloodstream infections by enhancing the sensitivity of conventional diagnostic approaches [41,42]. However, the absence of standardized protocols, the vast diversity of potentially pathogenic bacterial species residing in the oral cavity, and the presence of possible blood-derived PCR inhibitors represent significant challenges to the routine implementation of these methods [39]. Consequently, further studies are warranted to identify the bacterial species most frequently associated with bloodstream infections, endocarditis, and prosthetic valve infections, particularly in high-risk populations. These investigations should also aim to develop rapid, PCR-based diagnostic assays that could be applied in clinical settings where such infections are suspected, especially among immunocompromised patients (e.g., oncology patients and people living with HIV). Nevertheless, in the absence of species-specific breakpoints for *E. corrodens*, we reported MICs without categorical interpretation and selected therapy based on (i) low measured MICs, (ii) antibiotic CNS pharmacokinetics/pharmacodynamics, and (iii) clinical response. This approach aligns with EUCAST guidance for organisms lacking breakpoints and avoids potentially misleading extrapolation from phylogenetically related taxa [43]. However, interpretation against *Haemophilus influenzae* breakpoints has been applied given taxonomic proximity within the Neisseriaceae family in absence of EUCAST and CLSI criteria [44]. When interpreted according to *H. influenzae* criteria, our isolate would have been considered susceptible to β-lactams such as ceftriaxone and imipenem. Anyway, this strategy remains problematic: metabolic and resistance determinants differ between *H. influenzae* and *E. corrodens*, and neither CLSI nor EUCAST currently endorse such extrapolation. Therapeutic management of *E. corrodens* bacteremia in the setting of CNS infection requires antibiotics with both reliable activity against HACEK organisms and adequate CNS penetration [40]. Among β-lactams, third-generation cephalosporins—particularly ceftriaxone—are preferred due to potent bactericidal activity and favorable cerebrospinal fluid pharmacokinetics [45,46]. In our case, following identification of *E. corrodens* via MALDI-TOF MS and confirmation by 16S rRNA gene sequencing, intravenous ceftriaxone was administered at 2 g every 12 h, and clinical improvement was observed. In addition, neurosurgical drainage of subdural empyema is classically recommended to relieve mass effect and enhance microbial clearance [47]. However, in our case, multidisciplinary evaluation deemed surgery contraindicated. The patient’s advanced-stage nasopharyngeal carcinoma, severely impaired performance status, and progressive neurological deterioration suggested a very limited likelihood of meaningful recovery. Moreover, the anatomical extent of the empyema precluded less invasive drainage approaches, further increasing perioperative risks. Moreover, after reevaluating the particle therapy performed, the possibility of performing hadrontherapy was ruled out due to both the high risk of radiation necrosis and the impossibility of performing hadrontherapy with a palliative dose. In light of these considerations, the risks of surgical morbidity and poor functional outcome were judged to outweigh potential benefits. Therapeutic efforts thus focused on targeted antibiotic therapy while balancing patient comfort and prognosis. Subsequently, in accordance with shared decision-making principles, a palliative care approach was initiated.

## 6. Conclusions

The case presented herein illustrates how anatomical disruption and immunological imbalance—whether due to malignancy, local disease progression, or iatrogenic modulation of immune function—can synergistically facilitate microbial translocation from mucosal sites to the central nervous system and, ultimately, into the bloodstream. Emerging evidence suggests that ICIs, while enhancing anti-tumor immunity, may paradoxically compromise mucosal barrier integrity and microbiome homeostasis, increasing susceptibility to opportunistic infections. These alterations underscore the need for longitudinal studies evaluating the impact of ICIs on the oral and sinonasal microbiota, particularly in head and neck cancer populations. Monitoring shifts in microbial composition could serve as both a biomarker of immune perturbation and a predictive tool for infectious complications. Future research should focus on characterizing the immunological and microbiological interfaces altered by ICIs and establishing standardized protocols for the rapid identification and management of invasive infections caused by fastidious oral organisms such as *E. corrodens*.

## Figures and Tables

**Table 1:** The minimum inhibitory concentration (MIC) values for the *E. corrodens* organism isolated from the patient’s blood. No CLSI/EUCAST breakpoints are available for *E. corrodens*; MIC values are presented without categorical interpretation.

Antibiotic	MIC (µg/mL)
Ampicillin	≤0.064
Azithromycin	0.094
Ceftriaxone	0.006
Clindamycin	0.004
Imipenem	0.125
Meropenem	0.125
Penicillin G	0.094
Trimethoprim–Sulfamethoxazole	0.003

**Table 2:** *E. corrodens* head and neck infection with secondary bacteremia. IgG, immunoglobulin G; IV, intravenous; NA, not available; UK, United Kingdom.

Reference	Years	Clinical Presentation	Sex	Ethnicity	Age (Years)	Immunity State	Underlying Conditions	Initial Symptoms/Signs	Outcomes	Treatment	Resistance (MIC)	Bacteria Isolated	Identification	Country
Emmerson and Mills [17]	1978	Meningitis and right cerebellar abscess	Female	Caucasian	60-year-old	Immunocompetent	Several head and neck surgeries	Fever, generalized headache, anorexia, vomiting after meals, poor concentration, bizarre and erratic behavior and increasing drowsiness.	Died	Ampicillin 2 g/4 h IV for 10 days followed by oral amoxicillin 1 g/6 h and probenecid 500 mg b.d. for 1 week	No (disk diffusion)	*E. corrodens,* non-haemolytic *streptococcus*	Biochemical	UK
Zgheib A. et al. [18]	1992	Peritonsillar abscess and intrathoracic infection	Male	Caucasian	52	Immunocompetent	Oral antiseptic and surgical drainage of peritonsillar abscesses	Severe weakness, fever of 39.5 °C, dysphagia, mild retrosternal oppression	Survived	Erythromycin; Clindamycin in 400 mg IV 6-hourly; Gentamicin 5 mg/kg daily; Ticarcillin 5 g IV 6-hourly.	Clindamycin, amikacin (broth-dilution technique)	*Eikenella corrodens*	NA	Belgium
Dong and Gong [19]	2013	Chronic meningitis	Female	Asian	50-year-old	Immunocompetent	Alcoholism, smoking	Pharyngodynia, fever, headache	Survived	Ampicillin/sulbactam (12 g/day) and 0.4 g/day IV levofloxacin	Ceftazidime	*Eikenella corrodens*	NA	China
Sawyer et al. [20]	2015	Early onset neonatal sepsis and associated meningitis	Male	Caucasian	27-week	Immunosuppressed	Preterm labor	Respiratory distress, apnoea	Survived	ampicillin (100 mg/kg/dose every 12 h), gen- gentamicin (4.5 mg/kg/dose every 36 h), cefotaxime (50 mg/kg/dose every 12 h)	NA	*Eikenella corrodens*	NA	Texas
Yamagishi et al. [21]	2018	Lemierre’s syndrome	Male	Asian	44	Immunodepressed	IgG4-related ophthalmic disease	Dyspnoea, purpuric eruption	Survived	Meropenem; Clindamycin; Vancomycin; Ampicillin-Sulbactam; knee amputations	no	*Gemella bergeri, Eikenella corrodens*	ID Test HN-20 Rapidand16S rRNA gene sequencing	Japan

## Data Availability

The original contributions presented in this study are included in the article/Supplementary Materials. All relevant information is presented in the case report. Further inquiries can be directed at the corresponding authors.

## Supplementary Materials

The following supporting information can be downloaded at: https://www.mdpi.com/article/10.3390/microorganisms13092135/s1, Figure S1: Flow chart reporting the data selection and analysis method used in the systematic review. Fifty-four articles concerning *E. corrodens* infection according to the research strategy were examined. Forty-seven of these were excluded after the application of exclusion criteria. Among the seven remaining articles, two were excluded after reading the abstract or the full text. Five articles were included in the study.

## Abbreviations

The following abbreviations are used in this manuscript:

BSI	Bloodstream infection
CLSI	Clinical & Laboratory Standards Institute
CTLA-4	Cytotoxic T-Lymphocyte Antigen 4
CVC	Central venous catheter
EUCAST	European Committee on Antimicrobial Susceptibility Testing
HACEK	*Haemophilus species*, *Aggregatibacter actinomycetemcomitans*, *Cardiobacterium hominis*, *Eikenella corrodens*, *Kingella kingae*
HIV	Human immunodeficiency viruses
ICI	Immune checkpoint inhibitor
IgG	Immunoglobulin G
irAEs	Immune-related adverse events
MALDI-TOF MS	Matrix-Assisted Laser Desorption/Ionization Time-of-Flight Mass Spectrometry
MIC	Minimum Inhibitory Concentration
NCBI	National Center for Biotechnology Information
PCR	Polymerase chain reaction
PD-1	Programmed Cell Death Protein 1
rRNA	Ribosomal ribonucleic acid

## References

[1] Gajurel B.P., Giri S., Rayamajhi S., Khanal N., Bishowkarma S., Mishra A., Karn R., Rajbhandari R., Ojha R. (2023). Epidemiological and Clinical Characteristics of Central Nervous System Infections in a Tertiary Center: A Retrospective Study. Health Sci. Rep..

[2] Zimmer A.J., Burke V.E., Bloch K.C. (2016). Central Nervous System Infections. Microbiol. Spectr..

[3] Archibald L.K., Quisling R.G. (2013). Central Nervous System Infections. Textbook of Neurointensive Care.

[4] Brook I. (2003). Microbiology and Management of Deep Facial Infections and Lemierre Syndrome. ORL.

[5] Hu C.-Y., Lien K.-H., Chen S.-L., Chan K.-C. (2022). Risk Factors of Descending Necrotizing Mediastinitis in Deep Neck Abscesses. Medicina.

[6] Bandol G., Cobzeanu M.D., Moscalu M., Palade O.D., Moisii L., Severin F., Patrascanu E., Mocanu F., Roman A.I., Cobzeanu B.M. (2025). Deep Neck Infections: The Effectiveness of Therapeutic Management and Bacteriological Profile. Medicina.

[7] Zhou X., Wu Y., Zhu Z., Lu C., Zhang C., Zeng L., Xie F., Zhang L., Zhou F. (2025). Mucosal Immune Response in Biology, Disease Prevention and Treatment. Signal Transduct. Target. Ther..

[8] Patel K., Clifford D.B. (2014). Bacterial Brain Abscess. Neurohospitalist.

[9] Maniglia A.J., Goodwin W.J., Arnold J.E., Ganz E. (1989). Intracranial Abscesses Secondary to Nasal, Sinus, and Orbital Infections in Adults and Children. Arch. Otolaryngol. Head Neck Surg..

[10] Bodilsen J., Mariager T., Larsen L., Brandt C.T., Hansen B.R., Wiese L., Omland L.H., Nielsen H., Storgaard M., Larsen L. (2023). Brain Abscess Caused by Oral Cavity Bacteria: A Nationwide, Population-Based Cohort Study. Clin. Infect. Dis..

[11] Nguyen I., Urbanczyk K., Mtui E., Li S. (2020). Intracranial CNS Infections: A Literature Review and Radiology Case Studies. Semin. Ultrasound CT MRI.

[12] Rogacheva Y., Popova M., Lepik K., Kondakova E., Zalyalov Y., Stelmakh L., Volkova A., Nikolaev I., Goloshchapov O., Barhatov I. (2019). PS1282 Infectious Complications of Nivolumab Therapy in Relapsed/Refractory Hodgkin’s Lymphoma. HemaSphere.

[13] Ross J.A., Komoda K., Pal S., Dickter J., Salgia R., Dadwal S. (2021). Infectious Complications of Immune Checkpoint Inhibitors in Solid Organ Malignancies. Cancer Medicine.

[14] Ocaña-Guzmán R., Osorio-Pérez D., Chávez-Galán L. (2023). Opportunistic Infections and Immune-Related Adverse Events Associated with Administering Immune Checkpoint Inhibitors: A Narrative Review. Pharmaceuticals.

[15] Tezuka T., Takahashi N., Tokuyasu D., Azami S., Sekiguchi K., Takizawa T., Izawa Y., Nakahara J., Katsumata M. (2024). Cerebral Venous Thrombosis Mimicking Limbic Encephalitis. Intern. Med..

[16] Li L., Shi Y., Weng X. (2022). *Eikenella corrodens* Infections in Human: Reports of Six Cases and Review of Literatures. J. Clin. Lab. Anal..

[17] Emmerson A.M., Mills F. (1978). Recurrent Meningitis and Brain Abscess Caused by *Eikenella corrodens*. Postgrad. Med. J..

[18] Zgheib A., el Allaf D., Demonty J., Rorive G. (1992). Intrathoracic Infections with Bacteraemia due to *Eikenella corrodens* as a Complication of Peritonsillar Abscesses: Report of a Case and Review of the Literature. Acta Clin. Belg..

[19] Dong X.-Y., Gong L. (2013). Chronic Meningitis Caused by *Eikenella corrodens*. Kaohsiung J. Med. Sci..

[20] Sawyer C., Angelis D., Bennett R. (2015). *Eikenella corrodens* Sepsis with Cerebrospinal Fluid Pleocytosis in a Very Low Birth Weight Neonate. Case Rep. Pediatr..

[21] Yamagishi T., Hikone M., Sugiyama K., Tanabe T., Wada Y., Furugaito M., Arai Y., Uzawa Y., Mizushima R., Kamada K. (2018). Purpura Fulminans with Lemierre’s Syndrome Caused by *Gemella bergeri* and *Eikenella corrodens*: A Case Report. BMC Infect. Dis..

[22] Berge A., Morenius C., Petropoulos A., Nilson B., Rasmussen M. (2020). Epidemiology, Bacteriology, and Clinical Characteristics of HACEK Bacteremia and Endocarditis: A Population-Based Retrospective Study. Eur. J. Clin. Microbiol. Infect. Dis..

[23] Rodríguez-Rojas L., Suarez-López A., Cantón R., Ruiz-Garbajosa P. (2020). *Eikenella corrodens* Causing Deep-Seated Infections. Six-Year Experience in a University Hospital in Madrid. Enfermedades Infecc. Y Microbiol. Clínica.

[24] Tobinick E., Vega C.P. (2006). The Cerebrospinal Venous System: Anatomy, Physiology, and Clinical Implications. MedGenMed Medscape Gen. Med..

[25] Shiravand Y., Khodadadi F., Kashani S.M.A., Hosseini-Fard S.R., Hosseini S., Sadeghirad H., Ladwa R., O’Byrne K., Kulasinghe A. (2022). Immune Checkpoint Inhibitors in Cancer Therapy. Curr. Oncol..

[26] Petrelli F., Morelli A.M., Luciani A., Ghidini A., Solinas C. (2021). Risk of Infection with Immune Checkpoint Inhibitors: A Systematic Review and Meta-Analysis. Target. Oncol..

[27] Lombardi A., Saydere A., Ungaro R., Bozzi G., Viero G., Bandera A., Gori A., Mondelli M.U. (2022). Infectious Events in Patients Treated with Immune Checkpoint Inhibitors, Chimeric Antigen Receptor T Cells, and Bispecific T-Cell Engagers: A Review of Registration Studies. Int. J. Infect. Dis..

[28] Wang X., Wu Y., Hu W., Zhang J. (2025). Incidence and Risk Factors of Serious Infections Occurred in Patients with Lung Cancer Following Immune Checkpoint Blockade Therapy. BMC Cancer.

[29] Cosio T., Coniglione F., Flaminio V., Gaziano R., Coletta D., Petruccelli R., Dika E., Bianchi L., Campione E. (2023). Pyodermitis during Nivolumab Treatment for Non-Small Cell Lung Cancer: A Case Report and Review of the Literature. Int. J. Mol. Sci..

[30] Yin Q., Wu L., Han L., Xiao Z., Tong R., Lian L., Bai L., Bian Y. (2023). Immune-Related Adverse Events of Immune Checkpoint Inhibitors: A Review. Front. Immunol..

[31] Karam J.-D., Noel N., Voisin A.-L., Lanoy E., Michot J.-M., Lambotte O. (2020). Infectious Complications in Patients Treated with Immune Checkpoint Inhibitors. Eur. J. Cancer.

[32] Mannavola C.M., De Maio F., Marra J., Fiori B., Santarelli G., Posteraro B., Sica S., D’Inzeo T., Sanguinetti M. (2025). Bloodstream Infection by *Lactobacillus Rhamnosus* in a Haematology Patient: Why Metagenomics Can Make the Difference. Gut Pathog..

[33] Rajasekaran J.J., Krishnamurthy H.K., Bosco J., Jayaraman V., Krishna K., Wang T., Bei K. (2024). Oral Microbiome: A Review of Its Impact on Oral and Systemic Health. Microorganisms.

[34] Verheijden R.J., van Eijs M.V.M., Paganelli F.L., Viveen M.C., Rogers M.R.C., Top J., May A.M., van de Wijgert J.H., Suijkerbuijk K.P. (2025). Gut Microbiome and Immune Checkpoint Inhibitor Toxicity. Eur. J. Cancer.

[35] Ray K. (2017). Oral Microbiome Could Provide Clues to CRC. Nat. Rev. Gastroenterol. Hepatol..

[36] Liu J., Xu C., Wang R., Huang J., Zhao R., Wang R. (2025). Microbiota and Metabolomic Profiling Coupled with Machine Learning to Identify Biomarkers and Drug Targets in Nasopharyngeal Carcinoma. Front. Pharmacol..

[37] Sheng E., Ong Y., Chou Y., Then C.K. (2024). Interconnected Influences of Tumour and Host Microbiota on Treatment Response and Side Effects in Nasopharyngeal Cancer. Crit. Rev. Oncol. Hematol..

[38] Parahitiyawa N.B., Jin L.J., Leung W.K., Yam W.C., Samaranayake L.P. (2009). Microbiology of Odontogenic Bacteremia: Beyond Endocarditis. Clin. Microbiol. Rev..

[39] Castillo D.M., Sánchez-Beltrán M.C., Castellanos J.E., Sanz I., Mayorga-Fayad I., Sanz M., Lafaurie G.I. (2011). Detection of Specific Periodontal Microorganisms from Bacteraemia Samples after Periodontal Therapy Using Molecular-Based Diagnostics. J. Clin. Periodontol..

[40] Derosa L., Routy B., Desilets A., Daillère R., Terrisse S., Kroemer G., Zitvogel L. (2021). Microbiota-Centered Interventions: The next Breakthrough in Immuno-Oncology?. Cancer Discov..

[41] Avni T., Leibovici L., Paul M. (2010). PCR Diagnosis of Invasive Candidiasis: Systematic Review and Meta-Analysis. J. Clin. Microbiol..

[42] Jordana-Lluch E., Giménez M., Quesada M.D., Ausina V., Martró E. (2014). Improving the Diagnosis of Bloodstream Infections: PCR Coupled with Mass Spectrometry. BioMed Res. Int..

[43] European Committee on Antimicrobial Susceptibility Testing (2025). Guidance Document: What to Do When There Are No Breakpoints. Eucast. https://www.eucast.org/clinical_breakpoints_and_dosing/when_there_are_no_breakpoints.

[44] Luong N., Tsai J., Chen C. (2001). Susceptibilities of *Eikenella corrodens*, *Prevotella intermedia*, and *Prevotella nigrescens* Clinical Isolates to Amoxicillin and Tetracycline. Antimicrob. Agents Chemother..

[45] Nau R., Sorgel F., Eiffert H. (2010). Penetration of Drugs through the Blood-Cerebrospinal Fluid/Blood-Brain Barrier for Treatment of Central Nervous System Infections. Clin. Microbiol. Rev..

[46] Bodilsen J., D’Alessandris Q.G., Humphreys H., Iro M.A., Klein M., Last K., López-Montesinos I., Pagliano P., Sipahi O.R., Juan R.S. (2023). ESCMID Guidelines on Diagnosis and Treatment of Brain Abscess in Children and Adults. Clin. Microbiol. Infect..

[47] Brouwer M.C., Tunkel A.R., McKhann G.M., van de Beek D. (2014). Brain Abscess. N. Engl. J. Med..

