# Supplementary material for: Crossing the Barrier: Eikenella corrodens Bacteremia Following CNS Infection in a Patient Treated with Nivolumab—A Case Report and Literature Review
Source: Microorganisms. 2025 Sep 12;13(9):2135. doi: 10.3390/microorganisms13092135 (PMC12472742; doi:10.3390/microorganisms13092135)
Supplement: Supplementary file 1 [file microorganisms-13-02135-s001.zip › microorganisms-3830930-supplementary.pdf]

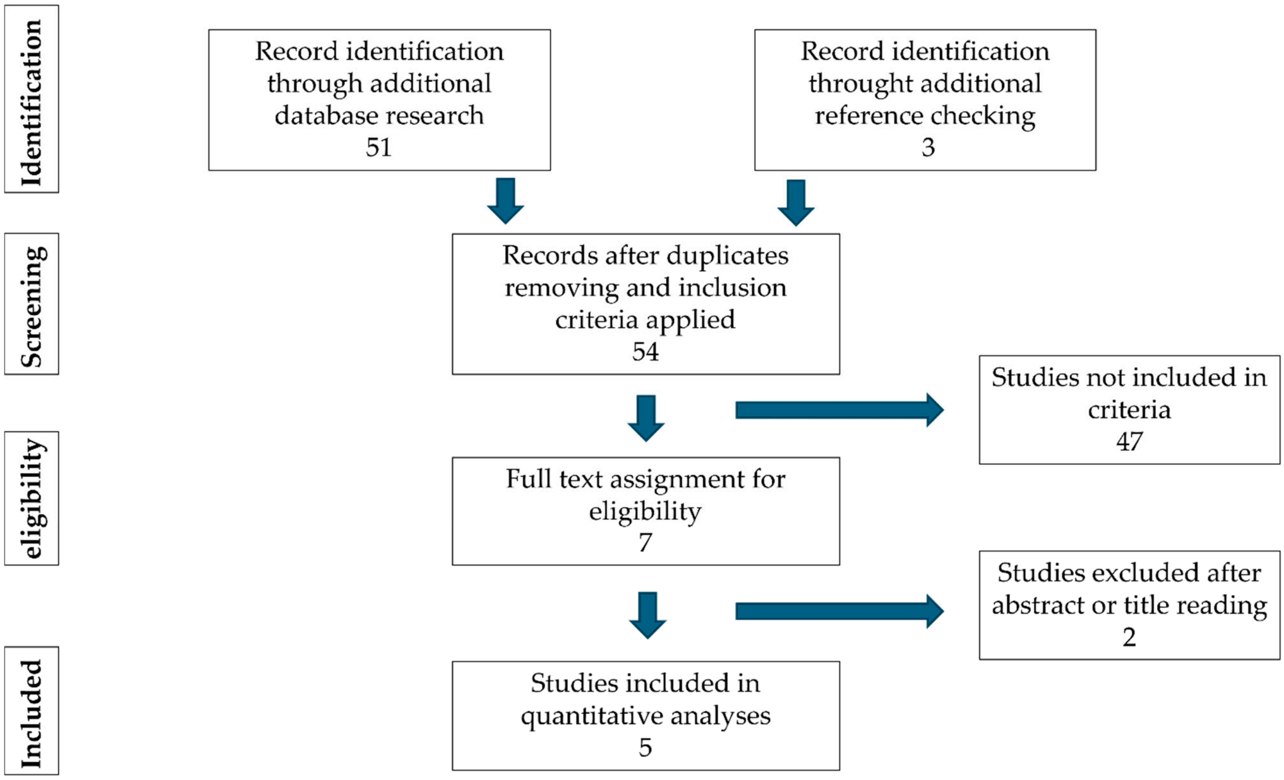

Figure S1. Flow chart reporting the data selection and analysis method used in the systematic review. Fifty-four articles concerning *E. corrodens* infection according to the research strategy were examined. Forty-seven of these were excluded after the application of exclusion criteria. Among the seven remaining articles, two were excluded after reading the abstract or the full text. Five articles were included in the study.
